# *QuickStats*: Age-Adjusted Death Rate* Among Adults Aged ≥65 Years, by Sex — United States, 1970–2022

**DOI:** 10.15585/mmwr.mm7326a3

**Published:** 2024-07-04

**Figure:**
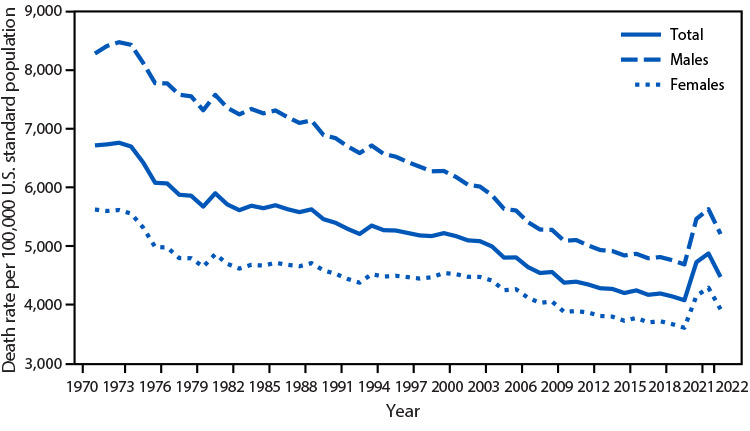
The age-adjusted death rate among adults aged ≥65 years declined from 6,717.6 per 100,000 standard population in 1970 to 4,073.8 in 2019. Death rates increased in 2020 and 2021 but then declined to 4,470.0 in 2022. The pattern was similar for males and females, although death rates for males were higher than those for females throughout the period 1970–2022.

**Supplementary Table**: https://stacks.cdc.gov/view/cdc/156507

**Source**: National Center for Health Statistics, National Vital Statistics System, Mortality Data, 1970–2022. https://www.cdc.gov/nchs/nvss/deaths.htm

